# Pre‐diagnosis lipid levels and mortality after obesity‐related cancer diagnosis in the Women's Health Initiative cardiovascular disease biomarker cohort

**DOI:** 10.1002/cam4.6266

**Published:** 2023-06-29

**Authors:** Gayane Hovsepyan, Ana Barac, Theodore M. Brasky, Aladdin H. Shadyab, Amy Lehman, Eric M. McLaughlin, Nazmus Saquib, Neil M. Iyengar, Robert A. Wild, Bette J. Caan, Pinkal Desai, Jennifer Beebe Dimmer, Cynthia A. Thomson, Michael S. Simon

**Affiliations:** ^1^ Wayne State University School of Medicine Detroit Michigan USA; ^2^ Scripps Green Internal Medicine Residency Program La Jolla California USA; ^3^ Georgetown University Washington District of Columbia USA; ^4^ Inova Heart and Vascular Institute Falls Church Virginia USA; ^5^ Comprehensive Cancer Center Ohio State University Columbus Ohio USA; ^6^ Herbert Wertheim School of Public Health and Human Longevity Science University of California, San Diego La Jolla California USA; ^7^ Center for Biostatistics Ohio State University Columbus Ohio USA; ^8^ College of Medicine Sulaiman AlRajhi University Al Bukayriyah Saudi Arabia; ^9^ Memorial Sloan Kettering Cancer Center New York New York USA; ^10^ University of Oklahoma Health Sciences Center Oklahoma City Oklahoma USA; ^11^ Division of Research Kaiser Permanente Medical Program of Northern California Oakland California USA; ^12^ Department of Oncology Weill Cornell Medical Center New York New York USA; ^13^ Department of Oncology Barbara Ann Karmanos Cancer Institute at Wayne Sate University Detroit Michigan USA; ^14^ Department of Health Promotion Sciences Mel & Enid Zuckerman College of Public Health and Arizona Cancer Center University of Arizona Tucson Arizona USA

**Keywords:** lipids, obesity related cancer, survival, women's health initiative

## Abstract

**Background:**

Published studies have demonstrated inconclusive relationships between serum lipid levels and mortality after cancer.

**Methods:**

The primary objective was to evaluate the relationship between fasting lipid levels and mortality after cancer. Data were obtained on baseline lipids and outcomes after cancer from 1263 postmenopausal women diagnosed with 13 obesity‐related cancers who were part of the Women's Health Initiative (WHI) lipid biomarkers cohort. Obesity‐related cancers included incident invasive cancers of the breast, colorectum, endometrium, esophagus (adenocarcinoma), kidney, liver, gallbladder, pancreas, ovaries, small intestine, thyroid, stomach, as well as multiple myeloma. Baseline lipid measurements included high‐density lipoprotein (HDL)‐cholesterol, low‐density lipoprotein (LDL)‐cholesterol, and non‐HDL‐cholesterol. Outcomes were all cause, cancer‐specific, and CVD mortality. Multivariable Cox proportional hazards models were used to measure associations between lipid levels and mortality (all cause, cancer, and CVD) after a cancer diagnosis, with lipids analyzed as continuous variables.

**Results:**

Among women with obesity‐related cancer, there were 707 deaths, of which 379 (54%) were due to cancer and 113 (16%) were due to CVD. Mean time from blood draw to cancer diagnosis was 5.1 years (range: 0.05–10 years). LDL‐C values above the 95th percentile were associated with higher risk of all‐cause mortality (*p* < 0.001), and cancer‐specific mortality (*p* < 0.001), but not mortality due to CVD. Non‐HDL‐C values above the 65th percentile were associated with higher risk of all‐cause mortality (*p* = 0.01) and mortality due to CVD (*p* = 0.003), but not cancer‐specific mortality (*p* = 0.37). HDL‐C values above the 95th percentile were associated with lower all‐cause mortality (*p* = 0.002), and above the 65th percentile with lower cancer‐specific mortality (*p* = 0.003), but no significant relationship with mortality due to CVD was observed.

**Conclusions:**

The relationship between pre‐diagnosis fasting lipid levels and mortality after cancer diagnosis is complex. These results suggest that improved lipid control through lifestyle and anti‐lipid medications could have a meaningful impact on outcomes after cancer.

## Graphical abstract

Baseline high LDL‐C and non‐HDL‐C values, have a potentially adverse impact, and high HDL‐C values have a potentially favorable impact on outcomes after cancer. These results suggest the importance of lipid control and lifestyle on improving long‐term cancer survivorship.

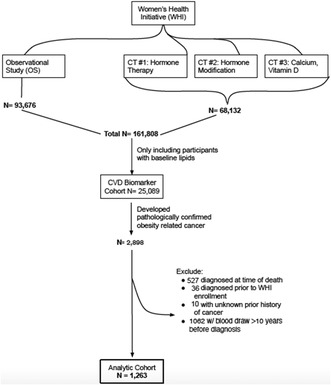

## 1 INTRODUCTION

Studies assessing the relationship between abnormal levels of lipid biomarkers such as total cholesterol (TC), high‐density lipoprotein cholesterol (HDL‐C), and low‐density lipoprotein cholesterol (LDL‐C), and cancer outcomes have yielded inconsistent results.^1^, ^2^, ^3^ In combination with waist circumference (WC) and clinical factors (e.g., hypertension (HTN), insulin resistance), routinely measured circulating lipids including LDL‐C, HDL‐C, and triglycerides (TG) provide a profile of an individual's metabolic health.^4^ There is an increasingly robust body of literature suggesting an interplay between metabolic health and cancer risk and outcomes, potentially by a common inflammatory pathway.^5^ More recently, non‐HDL‐C has been introduced as a variable of interest given its strongly positive association with long‐term risk of atherosclerotic cardiovascular disease.^6^ Some studies have found an impact of metabolic health on cancer risk, independent of body mass index.^7^, ^8^ A study using data from the Women's Health Initiative (WHI) reported associations between baseline lipids and incidence of several obesity‐related cancers in postmenopausal women; however, most associations were attenuated after the adjustment for clinical risk factors and statin use.^9^ Less information is available on the associations between individual lipid components and mortality after cancer.

To our knowledge only four published studies investigated the relationship between individual lipid components and mortality after cancer with resultant conflicting findings. High TC,^2^, ^3^, ^10^ high LDL‐C,^2^ and high HDL‐C^3^ were associated with a decreased risk of total cancer mortality in the cited references; however, other studies reported a higher rate of death from gynecologic cancer among women with high HDL‐C^2^ and a higher rate of total cancer mortality among individuals with higher HDL‐C.^1^

We used information on baseline lipid levels in a cohort of women in the WHI who were later diagnosed with 13 cancers established as obesity‐related in the US population^11^ for the primary objective of our research which was to evaluate the relationship between baseline lipid levels and mortality after cancer. Though there is a strong association between obesity and poor metabolic health, both of which are independently associated with certain cancers and dyslipidemia, the association between dyslipidemia and cancer risk, and especially outcomes after cancer is less clear. The WHI is an ideal setting for this analysis given the availability of baseline fasting lipid levels, adjudicated cancer diagnoses and mortality outcomes as well as detailed information on cardiometabolic and cardiovascular risk factors.

## 2 METHODS

### 2.1 Study population

**FIGURE 1:**
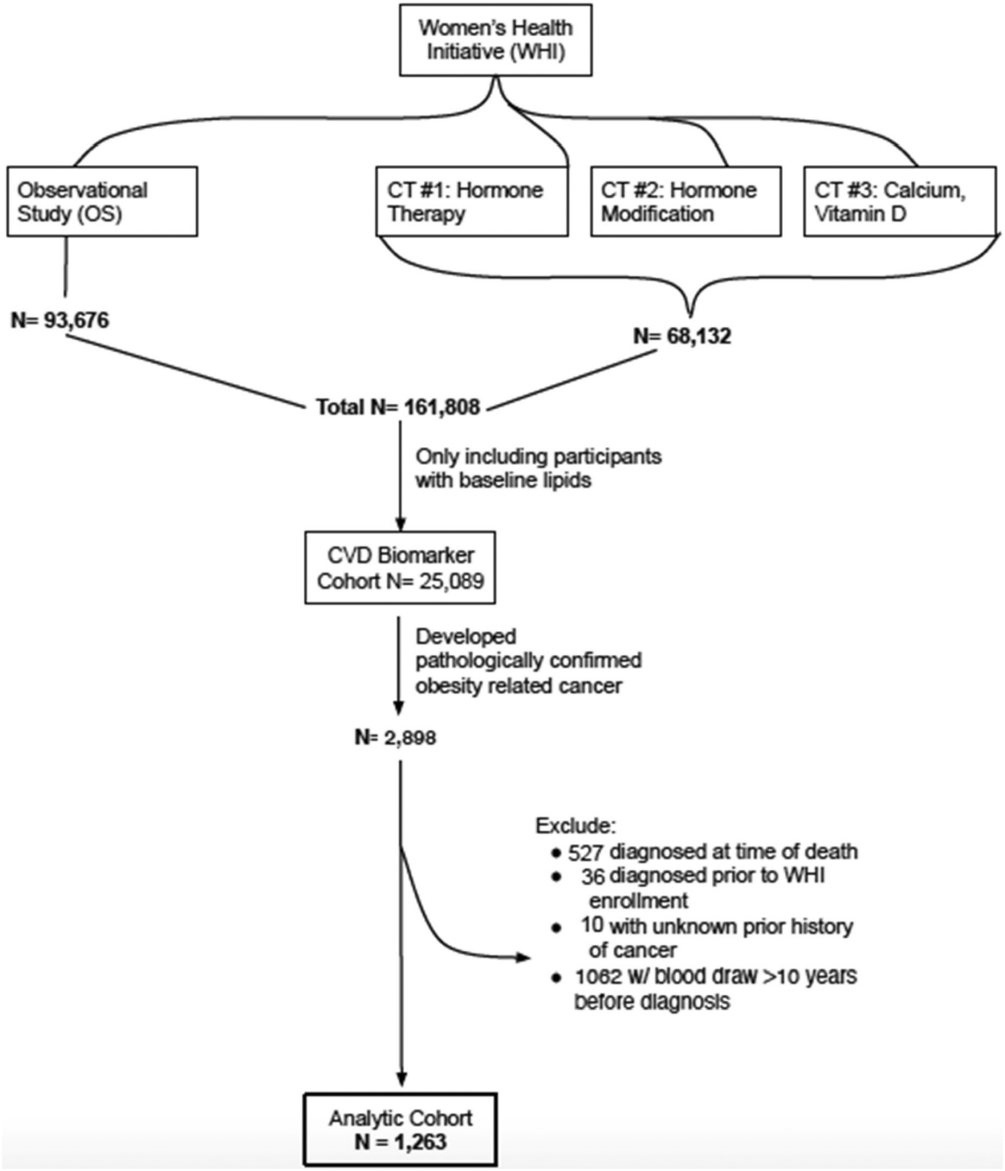
Consort diagram.

The WHI addressed significant causes of morbidity and mortality in postmenopausal women.^12^ The WHI includes four overlapping randomized clinical trials (CT) of combined estrogen plus progestin and unopposed estrogen hormone therapy (HT), dietary modification (DM), and calcium and vitamin D supplementation, as well as an observational cohort (OS).^12^ The cardiovascular disease (CVD) lipid biomarker cohort included WHI participants from all four CT arms of the WHI, who had baseline fasting lipid levels obtained at study entry (*n* = 25,089) (Figure 1). The goal of the CVD lipid biomarker cohort was to examine how baseline biomarkers relate to estrogen + progesterone or estrogen‐alone effects on CVD, and how biomarker changes might explain the impact of early hormone therapy on the risks of coronary heart disease (CHD), stroke, and venous thromboembolism.^13^

Our analytical sample included women who were part of the lipid biomarker cohort and who were diagnosed after study entry with any of 13 pathologically confirmed, incident, invasive, obesity‐related cancers including the breast, colorectum (CRC), endometrium, esophagus (adenocarcinoma), kidney, liver, gallbladder, pancreas, ovaries, small intestine, thyroid, stomach, as well as multiple myeloma. Among 2898 women diagnosed with an obesity‐related cancer and with baseline lipid information, we excluded 1062 women whose baseline blood draw was performed >10 years prior to their cancer diagnosis. This exclusion was made because of the longer duration of time between the lab measurement and the outcome of interest, and the possibility that baseline measures would not reflect exposure more proximal to the cancer diagnosis. Furthermore, our preliminary analysis demonstrated that women with blood draw >10 years prior to cancer diagnosis were much older (mean age 78.9 years with range 60.3–98.6 years compared to 70.8 years (range: 51.9, 87.2) for women with ≤10 years of time from baseline lab to cancer diagnosis) which could have potentially confounded our results. We also excluded 527 women diagnosed with cancer at the time of death, and 46 women with a baseline history of cancer, or missing history on cancer. These exclusions left a final analytic cohort of 1263 women with an obesity‐related invasive cancer diagnosis and a lipid measurement within 10 years prior to cancer diagnosis.

### 2.2 Lipid biomarkers

Plasma lipid biomarkers included in the analysis were LDL‐C, non‐HDL‐C (defined as TC minus HDL‐C), and HDL‐C. Blood specimens for CVD biomarker analyses were obtained after fasting for at least 8 hours for 99.8% of the WHI participants with blood measurements. The blood was centrifuged and subsequently serum and plasma specimens were frozen at −70 degrees centigrade and shipped to a central processing facility using dry ice.^14^ All lipid biomarker levels were measured in serum using a cholesterol oxidase method and HDL‐C using the HDL‐C plus third‐generation direct method at the same laboratory using standard protocols and QA/QC controls. LDL‐C was calculated with triglyceride (TG) <400 mg/dL by the Friedewald formula.^15^ Of note, TG levels did not exceed 400 mg/dL for our study population. For our analysis, we evaluated lipid biomarkers as continuous value variables.

### 2.3 Outcomes

The primary outcomes included all‐cause mortality, cancer‐specific mortality (for all obesity‐related cancers combined), and CVD mortality within 1–10 years post cancer diagnosis. The cause of death was determined by medical record review with both local and central adjudication by trained physician adjudicators and linkage to the National Death Index.^16^ Agreement between local versus central adjudication for death due to cancer (94%) and death due to CVD (73%) has been demonstrated in prior WHI analyses.^16^

### 2.4 Data collection

Participants completed an extensive series of standardized questionnaires at baseline that included information on sociodemographics, exposures, and medical history.^17^ Protocol‐driven, clinic‐based anthropometric measurements including height (cm), weight (kg), and hip and waist circumference (cm), were performed by trained study coordinators at participants' baseline clinic visits from which body mass index (BMI; kg/m^2^) and waist‐to‐hip ratio (WHR) were calculated. Information on nutrient intake was obtained from the WHI food frequency questionnaire.^18^ Covariates considered for statistical adjustment included enrollment in a specific CT, self‐identified race and ethnicity, years of education, pack‐years of smoking, treated hypertension, self‐reported diabetes,^14^ percentage of energy from dietary fat, estrogen and combined estrogen/progesterone use, physical activity (MET‐hours/week), and statin usage reported at study entry (yes or no). Information on age at cancer diagnosis was obtained by the annual WHI follow‐up questionnaire.

### 2.5 Statistical methods

Participants were followed for mortality outcomes with data included through September 30, 2020. Lab values were scaled to account for systematic ancillary study (batch) differences in measurements. Scaling was performed by fitting a simple linear model with the individual lab value (LDL‐C, non‐HDL‐C, and HDL‐C) as a function of the study, adjusted for age, race and ethnicity, BMI, physical activity, geographic region, and clinical trial arm. From that model, parameter estimates for the study were pulled out and used to shift lab values. As a result of shifting, models with lab values did not need to include adjustment for the ancillary study arm of the blood draw. As our study cohort consisted of data from multiple studies with different selection criteria within the WHI, analyses using inverse weighting were performed so that results would be more representative of all WHI CT women with invasive cancer. Probability weights were calculated from a logistic model with inclusion in the biomarker dataset as the outcome while including the following variables as covariates: age at screening, race and ethnicity, clinical trial arm, WHI Memory Study, Long Life Study, and Genomics and Randomized Trials Network study membership, hysterectomy status at baseline, and medical history at baseline (myocardial infarction, stroke, ventricular tachycardia, or diabetes).

Lipids were analyzed as continuous variables in the Cox proportional hazards models to examine the associations of LDL‐C, non‐HDL‐C, and HDL‐C values with all‐cause, cancer‐specific, and CVD mortality outcomes. For all models, the proportional hazards assumption was assessed using scaled Schoenfeld residual plots, and the linearity of continuous covariates was checked using martingale and deviance residual plots. Based on our linearity assessments, we considered restricted cubic splines to model a more flexible functional relationship between lipids and age. Models with three, four, and five knots were fit using percentiles suggested by Harrell^19^ with AIC/BIC criteria used for model selection. Hazard models were adjusted for age at cancer diagnosis, education, race, ethnicity, WHI CT or OS arm, whether the participant had a current health care provider, BMI, smoking status, alcohol intake, physical activity, and HT use. The baseline hazard was stratified by time from baseline lipid measurement to invasive cancer diagnosis, using quartiles to account for differences in measurement timing. We also tested for whether there was a different relationship between lipid and cancer outcomes for obese versus nonobese women by using interaction effects in the hazard models.

All analyses were performed using SAS version 9.4 (SAS Institute Inc.).

## 3 RESULTS

For women in the analytic cohort (*N* = 1263), the mean time from blood draw to cancer diagnosis was 5.1 years (range: 0.05–10 years), the mean time from baseline enrollment to last follow‐up was 14.4 years (range: 0.12–25.9 years), and the mean time from diagnosis of the first invasive cancer diagnosis to last follow‐up was 9.5 years (range: 0.003–24.3 years). Fifty‐five women (4.4%) were alive and lost to follow‐up at the time of the last contact. The five most common cancer types were breast (*n* = 620, 49.1%), colorectal (*n* = 265, 21.0%), endometrial (*n* = 86, 6.8%), pancreatic (*n* = 70, 5.5%), and ovarian (*n* = 60, 4.8%). Other cancer types included esophagus, gallbladder, kidney, liver, multiple myeloma, small intestine, stomach, and thyroid. Overall, 707 (56%) of the women died during follow‐up, of whom 377 (29.8%) died from invasive cancer representing 53% of all deaths. There were 113 women who died as a result of CVD. The top five causes of death due to cancer were colorectal (85), pancreatic (78), breast (71), ovarian (34), and multiple myeloma (32). The top five causes of death due to CVD were cerebral vascular (31), definite or possible CHD (47), pulmonary embolus (2), and other (33).

Selected descriptive baseline characteristics of study participants stratified by baseline median and interquartile ranges (IQR) for each lipid variable are listed in Table 1. The mean age at cancer diagnosis was 70.8 years (SD = 7.1). About 2/3 of the cohort consisted of non‐Hispanic White women and almost 2/3 had some college education. Regarding medical history, roughly three‐quarters of the women had a BMI within the overweight or obese classification, with the same proportion reporting past use of estrogen alone or estrogen and progesterone. The most common comorbidity was hypertension experienced by 41% of the cohort followed by diabetes in 12% of the cohort. A little over 50% of the women never smoked and 12% reported drinking on average seven or more alcoholic beverages per week.

**TABLE 1:** Distribution of lipid biomarker values by baseline characteristics in women with an obesity related cancer in the Women's Health Initiative CVD Biomarker cohort.

Characteristics	All women		Total cholesterol	Low‐density lipoprotein C^a^	High‐density lipoprotein C	Non‐HDLC
	*N*	%	Median (Q1–Q3)	Median (Q1–Q3)	Median (Q1–Q3)	Median (Q1–Q3)
Total	1263	100	226.8 [203.1–252.8]	149.3 [125.5–171.6]	50.3 [43.3–60.0]	173.8 [147.8–199.7]
Age at cancer diagnosis						
<55	85	6.7	217.1 [189.1–246.6]	134.9 [118.6–162.3]	52.3 [43.3–59.3]	158.3 [134.7–194.7]
55–59	180	14.3	226.9 [203.9–255.7]	152.4 [129.0–179.6]	50.3 [41.3–60.2]	175.7 [145.7–204.3]
60–69	599	47.4	225.8 [202.8–251.6]	148.5 [125.5–171.5]	50.0 [43.0–59.3]	174.3 [148.3–198.3]
≥70	399	31.6	230.8 [205.6–251.8]	151.5 [125.5–169.5]	52.0 [45.0–61.0]	173.8 [150.3–199.8]
Race						
White not of Hispanic origin	836	66.2	229.8 [204.8–253.8]	149.6 [125.5–171.5]	51.0 [44.0–60.0]	174.8 [149.8–200.8]
Black or African American	298	23.6	221.1 [198.1–250.1]	150.6 [123.1–176.6]	50.3 [42.3–60.3]	171.7 [140.3–197.7]
American Indian or Alaskan Native	3	0.2	178.6 [146.6–222.6]	110.6 [72.6–148.6]	52.3 [48.3–53.3]	130.3 [93.3–170.3]
Asian or Pacific Islander	23	1.8	214.6 [197.6–233.6]	138.1 [125.6–155.6]	51.3 [42.3–59.3]	161.3 [142.3–184.3]
Hispanic/Latino	100	7.9	224.1 [204.1–252.4]	143.6 [126.1–167.1]	47.3 [39.3–57.3]	172.3 [153.7–201.0]
Other	3	0.2	221.6 [178.6–225.6]	148.6 [105.6–148.6]	64.3 [56.3–65.3]	157.3 [113.3–169.3]
Education						
Less than some college	501	40.4	228.8 [203.8–254.8]	151.1 [126.5–176.1]	49.7 [42.0–58.3]	176.8 [148.8–205.8]
Some college	753	59.6	225.1 [203.1–248.8]	147.6 [125.5–169.1]	51.3 [44.3–60.3]	171.0 [146.7–195.0]
WHI study						
DM: intervention	245	19.4	225.1 [207.1–245.8]	150.3 [128.1–170.1]	50.3 [43.3–60.3]	174.0 [148.8–194.8]
DM: control	354	28.0	226.1 [198.1–253.6]	146.1 [123.5–173.1]	49.9 [42.3–60.3]	171.2 [146.3–199.8]
CT: not DM	664	52.6	228.2 [203.4–252.9]	149.6 [126.5–171.5]	51.0 [44.0–60.0]	174.8 [148.1–199.8]
Body mass index (BMI) kg/m^2^						
<25.0 kg/m^2^	277	22.0	220.1 [199.8–246.8]	140.1 [117.5–163.5]	58.0 [48.3–69.3]	159.8 [136.8–189.3]
25.0–29.9 kg/m^2^	392	31.1	230.8 [205.4–254.4]	152.3 [129.0–174.1]	51.0 [44.3–61.1]	175.8 [150.3–201.3]
30.0–34.9 kg/m^2^	333	26.4	226.1 [204.8–253.1]	149.5 [128.5–173.5]	49.0 [41.0–56.0]	176.7 [153.8–199.8]
≥35.0 kg/m^2^	257	20.4	226.8 [201.8–251.1]	152.1 [125.5–172.1]	46.3 [41.3–54.3]	176.8 [151.8–202.8]
Waist–hip ratio (WHR)						
<0.80	410	32.5	224.4 [201.8–246.8]	144.6 [122.5–169.5]	56.0 [48.0–66.3]	165.1 [139.3–192.3]
0.80–0.85	364	28.8	226.8 [203.8–248.7]	150.5 [125.5–171.1]	49.0 [42.0–57.7]	175.8 [151.2–196.5]
>0.85	488	38.7	229.1 [204.1–255.8]	152.1 [126.5–174.1]	48.0 [41.0–56.0]	177.8 [154.7–206.9]
Treated hypertension						
Never hypertensive	684	57.9	226.4 [203.8–251.8]	148.6 [126.5–171.5]	52.0 [45.0–61.0]	172.1 [146.7–197.3]
Treated hypertension	408	34.6	225.6 [199.9–250.1]	148.5 [121.5–170.1]	49.0 [42.0–56.6]	174.8 [146.8–198.8]
Untreated hypertension	89	7.5	234.8 [208.1–258.6]	154.3 [125.8–177.3]	52.0 [42.0–64.3]	180.3 [155.3–205.8]
Diabetes						
No	1112	88.0	227.1 [203.8–252.8]	149.6 [126.1–171.5]	51.3 [44.0–63.0]	173.3 [147.8–198.8]
Yes, treated	120	9.5	222.4 [196.4–250.1]	148.0 [116.1–173.5]	46.0 [39.2–52.2]	178.8 [143.0–204.3]
Yes, untreated	31	2.5	221.8 [192.8–251.8]	142.5 [114.5–176.5]	48.3 [39.0–54.0]	170.8 [142.3–201.8]
Smoking status						
Never smoker	657	52.6	227.8 [204.1–252.8]	150.6 [126.5–172.5]	51.0 [43.3–60.0]	175.0 [148.3–198.3]
Former smoker	491	39.3	226.8 [202.8–253.8]	148.1 [124.6–171.6]	51.0 [44.0–60.3]	172.8 [147.8–201.7]
Current smoker	100	8.0	221.4 [199.4–248.2]	148.6 [120.5–169.5]	47.3 [41.0–58.1]	170.8 [141.8–203.0]
Smoking pack‐years						
Never smoked	657	53.9	227.8 [204.1–252.8]	150.6 [126.5–172.5]	51.0 [43.3–60.0]	175.0 [148.3–198.3]
<5	149	12.2	226.8 [204.1–256.8]	149.6 [130.5–173.5]	50.3 [43.3–58.0]	175.7 [150.8–202.3]
5 to <20	164	13.5	223.5 [194.9–251.9]	147.1 [118.1–169.6]	51.0 [43.7–61.1]	170.0 [139.5–202.6]
≥20	249	20.4	224.8 [202.8–249.1]	147.6 [124.5–169.5]	50.0 [42.3–60.0]	172.7 [148.3–198.8]
Alcohol use						
Never drinker	172	13.8	226.6 [196.4–249.4]	151.6 [122.5–174.5]	48.0 [41.0–55.1]	176.5 [146.8–199.8]
Past drinker	282	22.6	228.8 [203.1–254.6]	150.6 [128.6–174.1]	48.3 [40.3–56.3]	178.8 [154.7–203.7]
<1 per month	179	14.3	226.8 [204.6–251.8]	149.6 [126.5–171.1]	49.3 [42.3–57.3]	174.7 [149.8–201.8]
<1 per week	241	19.3	224.8 [202.1–251.6]	146.5 [124.6–170.5]	50.3 [43.3–60.3]	171.3 [148.3–198.7]
1 to <7 per week	222	17.8	226.8 [204.8–249.8]	147.6 [123.5–169.5]	53.6 [46.3–63.0]	171.2 [144.3–194.7]
>7 per week	152	12.2	225.9 [204.2–253.7]	148.0 [125.5–166.0]	56.8 [49.0–68.5]	167.3 [142.3–192.3]
Physical activity						
0–0.75 MET‐hours/week	294	24.8	224.7 [204.8–250.1]	150.6 [124.1–173.1]	49.3 [42.0–57.0]	174.8 [150.8–201.7]
1–5.9	298	25.1	228.1 [200.0–254.1]	148.5 [122.6–171.5]	50.3 [44.0–59.3]	173.8 [143.3–200.8]
6–13.7	304	25.6	225.9 [199.8–248.4]	147.6 [125.5–172.5]	50.0 [43.0–60.0]	172.8 [145.3–198.6]
13.8–90.2	291	24.5	226.8 [205.1–253.1]	149.6 [127.6–169.5]	53.0 [44.3–63.0]	172.8 [148.7–196.8]
HRT usage						
Estrogen	336	26.6	231.8 [207.3–256.9]	152.6 [127.8–174.5]	49.3 [43.0–57.3]	181.8 [154.8–207.8]
Estrogen and progesterone	600	47.5	225.7 [202.1–251.9]	148.5 [126.5–171.3]	51.0 [44.7–60.0]	172.8 [147.8–198.8]
Not randomized to HRT	327	25.9	222.6 [198.1–248.1]	146.6 [121.6–170.1]	50.3 [42.3–61.3]	170.3 [140.7–194.3]
Statin use						
No	1160	91.8	228.1 [203.1–253.1]	150.3 [126.1–173.1]	51.0 [43.3–60.3]	174.7 [147.8–200.8]
Yes	103	8.2	218.8 [197.8–238.8]	139.1 [116.3–153.6]	48.0 [41.0–54.0]	170.7 [147.3–186.8]

^a^ 17 patients had no data for LDL‐C.

In comparing the oldest women (≥ age 70) to the youngest women (< age 55), older women tended to have higher values for TC, LDL‐C, and non‐HDL‐C, but no apparent difference in HDL‐C values by age. Women with prior estrogen use had higher values of TC, LDL‐C, and non‐HDL‐C. Women with higher educational levels, lower lean body mass, and higher levels of physical activity had higher HDL‐C levels.

**FIGURE 2:**
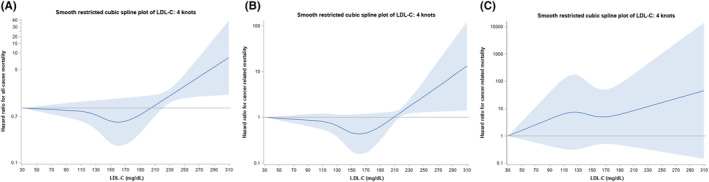
(A) Cox hazard ratios with 95% confidence bands for low‐density lipoprotein cholesterol and all‐cause mortality in the Women's Health Initiative lipid biomarker cohort. (B) Cox hazard ratios with 95% confidence bands for low‐density lipoprotein cholesterol and cancer‐specific mortality in Women's Health Initiative lipid biomarker cohort. (C) Cox hazard ratios with 95% confidence bands for low‐density lipoprotein cholesterol and cardiovascular disease mortality in the Women's Health Initiative lipid biomarker cohort.

**FIGURE 3:**
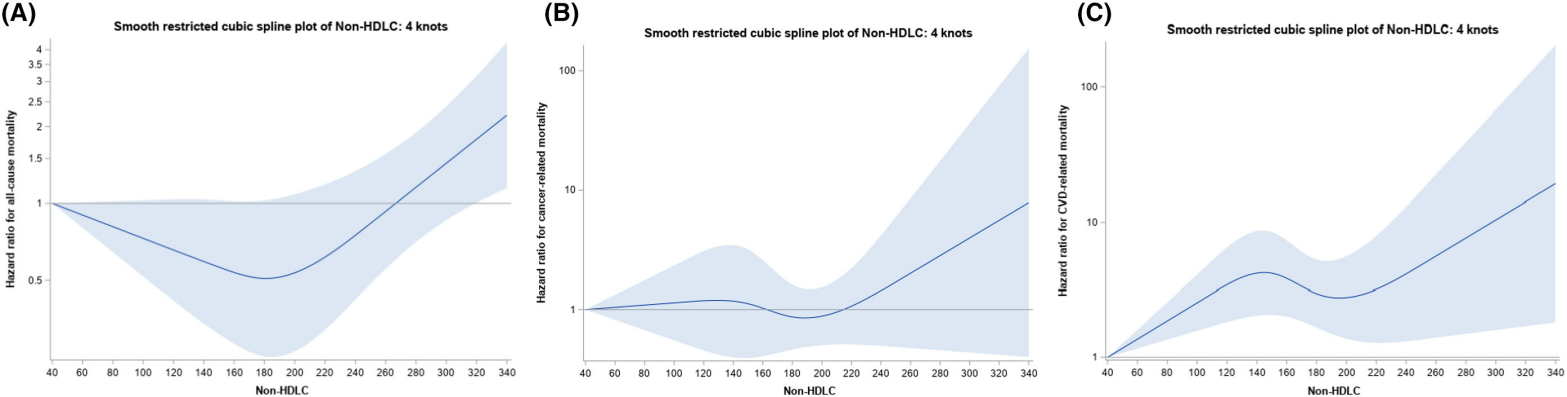
(A) Cox hazard ratios with 95% confidence bands for non–high‐density lipoprotein cholesterol and all‐cause mortality in the Women's Health Initiative lipid biomarker cohort. (B) Cox hazard ratios with 95% confidence bands for non‐HDLC and cancer‐specific mortality in the Women's Health Initiative lipid biomarker cohort. (C) Cox hazard ratios with 95% confidence bands for non–high‐density lipoprotein cholesterol and cardiovascular disease mortality in the Women's Health Initiative lipid biomarker cohort.

Figure 2A–C show the continuous variable survival analyses of LDL‐C and all‐cause mortality, cancer‐specific mortality, and mortality due to CVD. There was a weak but significant increase in all‐cause mortality above the level of 215 mg/dL (*p* < 0.001), and cancer‐specific mortality above the level of 245 mg/dL (both 95th percentile knots, *n* = 49 above 215 mg/dL, *n* = 8 above 245 mg/dL) (*p* < 0.001). There was no significant relationship between LDL‐C and mortality due to CVD. With regards to non‐HDL‐C, as shown in Figure 3, there was a small negative association with all‐cause mortality as the confidence band barely covered 1 up to around the 65th percentile, after which the risk of all‐cause mortality increased (*p* = 0.01). However, as shown in Figure 3B, there was no association with cancer‐specific mortality (*p* = 0.37). There was an increased risk for CVD mortality across increasing levels of non‐HDL‐C (3c) (*p* = 0.003).

**FIGURE 4:**
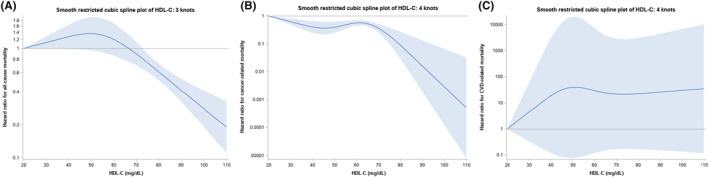
(A) Cox hazard ratios with 95% confidence bands for high‐density lipoprotein cholesterol and all‐cause mortality in the Women's Health Initiative lipid biomarker cohort. (B) Cox hazard ratios with 95% confidence bands for high‐density lipoprotein and cancer‐specific mortality in the Women's Health Initiative lipid biomarker cohort. (C) Cox hazard ratios with 95% confidence bands for high‐density lipoprotein cholesterol and cardiovascular disease mortality in the Women's Health Initiative lipid biomarker cohort.

The analysis of the associations of HDL‐C showed a 15% decreased risk of overall mortality (HR 0.85, 95% CI, 0.83–0.88; *p* < 0.001), while a 25 mg/dL increase in HDL‐C yielded a 20% decreased risk of overall mortality (HR 0.80, 95% CI: 0.77, 0.85; *p* < 0.001) (Figure 4). The restricted cubic spline plots suggested that these hazards may be driven by the upper 95th percentile of values as there was no strong change in hazard until the 95th percentile. For cancer‐specific mortality, there was a significantly lower risk observed with HDL‐C values above the 65th percentile which correlated to the approximate value of 60 mg/dL (*p* = 0.003) (Figure 4B). There was no significant relationship for mortality due to CVD (Figure 4C).

Table 2A–C shows the models with significant interaction effects between TC, HDL‐C, and non‐HDL‐C lipid quartiles, and obesity on all‐cause mortality. Just under half of our cohort was obese with a BMI >30 (*N* = 590, 46.9%). Mortality risk tended to decrease in higher TC quartiles in nonobese women, while risk tended to be highest in the lowest and highest quartiles of obese women (Table 2A). When assessing HDL‐C, nonobese women tended to have lower mortality risk than obese women, while also decreasing at higher HDL‐C quartiles in nonobese women (Table 2B). Risks fluctuated in non‐monotone directions across HDL‐C quartiles in obese women. Mortality trends across non‐HDL‐C quartiles were also of a non‐monotone direction, despite the significant effect modification from obesity (Table 2C).

**TABLE 2:** Hazard ratios and 95% confidence intervals for the relationship between lipid biomarkers and cancer outcomes in the Women's Health Initiative lipid biomarker cohort with effect modification of body mass index.

(A) Total cholesterol and all‐cause mortality—interaction *p*‐value <0.001			
TC	HR	95% CI	*p*‐value
Obese women (BMI > 30)			
Q2 vs. Q1	0.54	0.26–1.11	0.09
Q3 vs. Q1	0.57	0.34–0.93	0.03
Q4 vs. Q1	1.10	0.93–1.30	0.28
Nonobese women (BMI < 30)			
Q2 vs. Q1	1.31	1.02–1.66	0.03
Q3 vs. Q1	1.04	0.87–1.25	0.66
Q4 vs. Q1	0.87	0.75–1.00	0.046

(B) High‐density lipoprotein and all‐cause mortality—interaction *p*‐value <0.001			
HDL‐C	HR	95% CI	*p*‐value
Obese women (BMI > 30)			
Q2 vs. Q1	1.13	0.66–1.94	0.65
Q3 vs. Q1	0.89	0.66–1.21	0.47
Q4 vs. Q1	1.54	0.75–3.18	0.24
Nonobese women (BMI < 30)			
Q2 vs. Q1	1.25	1.20–1.31	<0.001
Q3 vs. Q1	0.72	0.56–0.93	0.01
Q4 vs. Q1	0.64	0.41–0.99	0.045

(C) Non‐HDL‐C and all‐cause mortality—interaction *p*‐value <0.001			
Non‐HDL‐C	HR	95% CI	*p*‐value
Obese women (BMI > 30)			
Q2 vs. Q1	0.61	0.28–1.30	0.20
Q3 vs. Q1	0.59	0.26–1.31	0.19
Q4 vs. Q1	1.02	0.72–1.45	0.92
Nonobese women (BMI < 30)			
Q2 vs. Q1	0.59	0.44–0.78	<0.001
Q3 vs. Q1	0.86	0.71–1.03	0.10
Q4 vs. Q1	0.99	0.88–1.11	0.82

## 4 DISCUSSION

We used data from the WHI's lipid biomarker cohort to examine the relationship between baseline fasting lipid values and mortality after cancer. To our knowledge, this is the only report evaluating fasting lipid values and mortality after cancer analyzing lipids as continuous value variables. We found a significant relationship between higher LDL‐C and increased risk of all‐cause and cancer‐specific mortality, largely driven by LDL‐C values above the 95th percentile, which if true will only impact a small proportion of women with cancer and very high lipid values. At the same time, there was a small negative association for non‐HDL‐C with all‐cause mortality as the confidence band barely covered 1 up to around the 65th percentile, after which the risk of all‐cause mortality increased as it did for LDL‐C. There was also a significant relationship between higher HDL‐C and lower cancer‐specific mortality for HDL values above the 65th percentile. The potential protective impact of high HDL on cancer‐specific mortality could potentially impact a larger proportion of women at risk. Lastly, despite fewer deaths from CVD in this cohort, we found a significant positive relationship between non‐HDL‐C and CVD mortality. We also found significant effect modification by obesity on the association of all examined lipid values and all‐cause mortality. Consistent with our findings, data from the EPIC‐Heidelberg cohort (*N* = 2739 of which 1632 had a cancer diagnosis) showed that elevated HDL‐C was associated with a reduction in total cancer mortality. The study population in the EPIC‐Heidelberg cohort was different than the WHI in that it included both men and women and a younger age group (ages 35–65 years).^3^ Consistent with our findings, results from the Women's Health Study, which evaluated HDL‐C as quartiles, demonstrated higher quartiles of HDL‐C were associated with a lower risk of total cancer mortality.^1^ In contrast, a study from the Lipids Research Clinic Program (2753 men and 2476 women aged 40–79) found a significant inverse association between LDL‐C with total cancer mortality among men, as well as a positive association between HDL‐C and death from gynecologic cancers.^2^ In the Lipids Research Clinic cohort, there was no significant relationship between HDL‐C and total cancer mortality in either men or women. It should be noted that in the Lipids Research Clinic Program study, the number of outcomes was small with only 79 reported cancer deaths in men and 65 in women.^2^

In our analysis we evaluated lipid levels as continuous value variables to better understand a potentially complex relationship between lipid levels and cancer outcomes, and to better identify signals missed in the quartile analyses from other studies. In our analysis, the continuous models were significant for the most part at extreme values (>95th percentile) while significant for HDL‐C and cancer‐specific mortality at the 65th percentile. While not conclusive of a cause‐and‐effect relationship, the relationship between LDL‐C and increased cancer mortality risk, as well as the potential protective effect of HDL‐C are consistent with relationships seen for cardiovascular health.^20^

In the cubic spline plots, the relationships for non‐HDL versus all‐cause mortality, as well as LDL‐C versus cancer‐specific mortality, there was a trough in the middle range of lipid values. This is similar to the U‐shaped relationships between LDL‐C and mortality outcomes described in the literature.^21^ HDL‐C versus all‐cause mortality, however, had the inverse shape, with mortality peaking in the middle range of values. This is contrary to a growing body of literature that suggests that HDL‐C might have harmful mortality effects in adults without cancer at the upper and lower range of values.^22^, ^23^, ^24^, ^25^

Potential mechanisms to explain the finding that lower risk of mortality was associated with higher HDL‐C are multifactorial. Low HDL‐C has been associated with increased likelihood of tumor metastasis.^26^ In addition, HDL is under investigation as a potential modulatory factor that reduces hypoxia‐mediated angiogenesis and inhibits inflammatory‐driven neovascularization, mechanisms necessary for cancer growth, and tumor metastasis.^27^ There is also evidence suggesting that HDL‐C has anti‐inflammatory and anti‐oxidative properties that may play a role in other inflammatory related processes such as cancer.^28^, ^29^, ^30^ Our study raises a further research question as to why HDL‐C is more strongly associated with mortality outcomes than LDL‐C. The answer may lie with the nature of lipid physiology, such that most medical interventions which seek to increase HDL‐C levels also result in reduced LDL‐C, triglycerides, BMI, and other markers of metabolic health.

Strengths of our analysis include the large sample size with baseline fasting lipid values and long duration of follow‐up for mortality outcomes. Limitations include the fact that we only studied postmenopausal women and our data cannot be generalized to other age groups or men. Furthermore, we only had information on baseline lipid values, and our data did not allow us to analyze changes in lipid profile over time. The average 5.1 years between lipid measurement and cancer diagnosis may also limit interpretation in that it is not clear what is the most informative time point prior to diagnosis wherein values may predict risk. In addition, we only adjusted for statin use at baseline because of the varying time points in which information on statin use was available. There is no information on statin dose or compliance with statins available in the WHI population. Another limitation of our study is that we analyzed all 13 obesity‐related cancers in a combined analysis. Given that the complex relationship between obesity, lipids, metabolic health, and cancer may vary by cancer site, it would be prudent to analyze each cancer separately as well; however, we lacked the power to do this. Lastly, we did not have information on other medical interventions such as cancer treatment that could have affected outcomes, although it is assumed that most participants in a large clinical trial received standard of care treatment for their cancers.

In conclusion, in an evaluation of the relationship between lipid levels and mortality after cancer, we found that very high LDL‐C and non‐HDL‐C are associated with greater all‐cause and cancer‐specific mortality in a sample of postmenopausal women who developed obesity‐related cancers. However, the clinical relevance of these findings is limited in that lipid levels mostly above the 95th percentile are rarely seen in humans. Higher HDL‐C was inversely associated with all‐cause and cancer‐specific mortality, suggesting that interventions (lifestyle and medications) to increase HDL‐C may serve to improve mortality outcomes after an obesity‐related cancer diagnosis.

## AUTHOR CONTRIBUTIONS

**Gayane Hovsepyan:** Conceptualization (lead); methodology (equal); writing – original draft (lead); writing – review and editing (lead). **Ana Barac:** Conceptualization (equal); methodology (equal); writing – original draft (supporting); writing – review and editing (supporting). **Theodore M. Brasky:** Conceptualization (equal); methodology (equal); writing – original draft (supporting); writing – review and editing (supporting). **Aladdin H Shadyab:** Conceptualization (equal); methodology (equal); writing – original draft (equal); writing – review and editing (equal). **Amy Lehman:** Conceptualization (equal); formal analysis (lead); methodology (lead); writing – original draft (supporting); writing – review and editing (supporting). **Eric McLaughlin:** Formal analysis (lead); methodology (lead); writing – review and editing (supporting). **Nazmus Saquib:** Conceptualization (equal); writing – review and editing (equal). **Neil M. Iyengar:** Conceptualization (lead); methodology (equal); writing – review and editing (equal). **Robert A Wild:** Conceptualization (equal); methodology (equal); writing – original draft (equal); writing – review and editing (equal). **Bette Caan:** Conceptualization (equal); writing – original draft (equal); writing – review and editing (equal). **Pinkal Desai:** Conceptualization (equal); writing – review and editing (equal). **Jennifer Beebe‐Dimmer:** Writing – review and editing (supporting). **Cynthia A Thomson:** Conceptualization (equal); writing – original draft (equal); writing – review and editing (equal). **Michael Simon:** Conceptualization (lead); resources (equal); writing – original draft (lead); writing – review and editing (lead).

## IRB/ETHICS COMMITTEE

The data from this manuscript were based on data previously collected and analyzed in the Women's Health initiative (WHI) database. There was no requirement for further IRB or Ethics Committee approval for the analysis that was completed. Written informed consent was previously obtained for the WHI, but again no further informed consent was needed for completion of an analysis based on previously collected data.

## Data Availability

Data available on request from the authors.

## References

[1] Chandler PD , Song Y , Lin J , et al. Lipid biomarkers and long‐term risk of cancer in the Women's health study. Am J Clin Nutr. 2016;103(6):1397‐1407. doi:10.3945/AJCN.115.124321 27099252 PMC4880994

[2] Cowan LD , O'connell DL , Criqui MH , Barrett‐connor E , Bush TL , Wallace RB . Cancer mortality and lipid and lipoprotein levels. Lipid research clinics program mortality follow‐up study. Am J Epidemiol. 1990;131(3):468‐482. doi:10.1093/OXFORDJOURNALS.AJE.A115521 2301356

[3] Katzke VA , Sookthai D , Johnson T , Kühn T , Kaaks R . Blood lipids and lipoproteins in relation to incidence and mortality risks for CVD and cancer in the prospective EPIC‐Heidelberg cohort. BMC Med. 2017;15(1):218. doi:10.1186/S12916-017-0976-4 29254484 PMC5735858

[4] Busnello FM , Santos ZE d A , Pontin B . Lipids and metabolic syndrome. In: Handbook of Lipids in Human Function: Fatty Acids. AOCS Press; 2016:543‐556.

[5] Koene RJ , Prizment AE , Blaes A , Konety SH . Shared risk factors in cardiovascular disease and cancer. Circulation. 2016;133(11):1104‐1114. doi:10.1161/CIRCULATIONAHA.115.020406 26976915 PMC4800750

[6] Brunner FJ , Waldeyer C , Ojeda F , et al. Application of non‐HDL cholesterol for population‐based cardiovascular risk stratification: results from the multinational cardiovascular risk consortium. Lancet. 2019;394(10215):2173‐2183. doi:10.1016/S0140-6736(19)32519-X 31810609 PMC6913519

[7] Iyengar NM , Arthur R , Manson JE , et al. Association of Body fat and Risk of breast cancer in postmenopausal women with Normal body mass index: a secondary analysis of a randomized clinical trial and observational study. JAMA Oncol. 2019;5(2):155‐163. doi:10.1001/JAMAONCOL.2018.5327 30520976 PMC6439554

[8] Liang X , Margolis KL , Hendryx M , et al. Metabolic phenotype and risk of colorectal cancer in Normal‐weight postmenopausal women. Cancer Epidemiol Biomark Prev. 2017;26(2):155‐161. doi:10.1158/1055-9965.EPI-16-0761 PMC5301805 28148595

[9] Kabat GC , Kim MY , Chlebowski RT , Vitolins MZ , Wassertheil‐Smoller S , Rohan TE . Serum lipids and risk of obesity‐related cancers in postmenopausal women. Cancer Causes Control. 2018;29(1):13‐24. doi:10.1007/S10552-017-0991-Y 29197994

[10] Törnberg SA , Holm LE , Carstensen JM , Eklund GA . Cancer incidence and cancer mortality in relation to serum cholesterol. J Natl Cancer Inst. 1989;81(24):1917‐1921. doi:10.1093/jnci/81.24.1917 2593170

[11] Steele CB , Thomas CC , Henley SJ , et al. Vital signs: trends in incidence of cancers associated with overweight and obesity — United States, 2005–2014. MMWR Morb Mortal Wkly Rep. 2017;66:1052‐1058. doi:10.15585/mmwr.mm6639e1 28981482 PMC5720881

[12] About WHI . About WHI . n.d. Accessed November 14, 2021. https://www.whi.org/page/about‐whi

[13] W6 ‐ HT CVD Biomarker Case‐Control Study of CHD, Stroke, and VTE . W6 ‐ HT CVD Biomarker Case‐Control Study of CHD, Stroke, and VTE . n.d. Accessed November 14, 2021. https://sp.whi.org/researchers/data/WHIStudies/StudySites/W6/pages/home.aspx

[14] Margolis KL , Qi L , Brzyski R , et al. Validity of diabetes self‐reports in the Women's Health Initiative: comparison with medication inventories and fasting glucose measurements. Clin Trials. 2008;5(3):240‐247. doi:10.1177/1740774508091749 18559413 PMC2757268

[15] WHI Extension Study CVD Biomarker Lab Methods . WHI Extension Study CVD Biomarker Lab Methods . n.d. Accesssed November 14, 2021. https://sp.whi.org/researchers/_layouts/15/WopiFrame.aspx?sourcedoc=/researchers/Documents/WHIExtensionStudyCVDBiomarkerLabMethods.pdf&action=default

[16] Curb JD , Mctiernan A , Heckbert SR , et al. Outcomes ascertainment and adjudication methods in the Women's Health Initiative. Ann Epidemiol. 2003;13(9 Suppl):S122‐8. doi:10.1016/S1047-2797(03)00048-6 14575944

[17] Anderson GL , Manson J , Wallace R , et al. Implementation of the women's health initiative study design. Ann Epidemiol. 2003;13(9):S5‐S17. doi:10.1016/S1047-2797(03)00043-7 14575938

[18] Form 60 ‐ Food Frequency Questionnaire ‐ Nutrients . Form 60 ‐ Food Frequency Questionnaire ‐ Nutrients . n.d. Accessed February 2, 2022. https://www.whi.org/dataset/290

[19] Harrell FE . Regression Modeling Strategies: with Applications to Linear Models, Logistic Regression, and Survival Analysis. Springer‐Verlag; 2010.

[20] Jung E , Kong SY , Ro YS , Ryu HH , Shin SD . Serum cholesterol levels and risk of cardiovascular death: a systematic review and a dose‐response meta‐analysis of prospective cohort studies. Int J Environ Res Public Health. 2022;19(14):8272. doi:10.3390/ijerph19148272 35886124 PMC9316578

[21] Johannesen CDL , Langsted A , Mortensen MB , Nordestgaard BG . Association between low density lipoprotein and all cause and cause specific mortality in Denmark: prospective cohort study. BMJ. 2020;371:m4266. doi:10.1136/BMJ.M4266 33293274 PMC7722479

[22] Allard‐Ratick M , Khambhati J , Topel M , Sandesara P , Sperling L , Quyyumi A . 50Elevated HDL‐C is associated with adverse cardiovascular outcomes. Eur Heart J. 2018;39(suppl_1):ehy564.50. doi:10.1093/EURHEARTJ/EHY564.50

[23] Hirata A , Sugiyama D , Watanabe M , et al. Association of extremely high levels of high‐density lipoprotein cholesterol with cardiovascular mortality in a pooled analysis of 9 cohort studies including 43,407 individuals: the EPOCH‐Japan study. J Clin Lipidol. 2018;12(3):674‐684.e5. doi:10.1016/J.JACL.2018.01.014 29506864

[24] Ko DT , Alter DA , Guo H , et al. High‐density lipoprotein cholesterol and cause‐specific mortality in individuals without previous cardiovascular conditions: the CANHEART study. J Am Coll Cardiol. 2016;68(19):2073‐2083. doi:10.1016/J.JACC.2016.08.038 27810046

[25] Madsen CM , Varbo A , Nordestgaard BG . Extreme high high‐density lipoprotein cholesterol is paradoxically associated with high mortality in men and women: two prospective cohort studies. Eur Heart J. 2017;38(32):2478‐2486. doi:10.1093/EURHEARTJ/EHX163 28419274

[26] Fiorenza AM , Branchi A , Sommariva D . Serum lipoprotein profile in patients with cancer. A comparison with non‐cancer subjects. Int J Clin Lab Res. 2000;30(3):141‐145. doi:10.1007/S005990070013 11196072

[27] Tan JTM , Ng MKC , Bursill CA . The role of high‐density lipoproteins in the regulation of angiogenesis. Cardiovasc Res. 2015;106(2):184‐193. doi:10.1093/CVR/CVV104 25759067

[28] Inacio Pinto N , Carnier J , Oyama LM , et al. Cancer as a proinflammatory environment: metastasis and cachexia. Mediat Inflamm. 2015;2015:791060. doi:10.1155/2015/791060 PMC4609868 26508818

[29] Kuvin JT , Karas RH . The effects of LDL reduction and HDL augmentation on physiologic and inflammatory markers. Curr Opin Cardiol. 2003;18(4):295‐300. doi:10.1097/00001573-200307000-00009 12858128

[30] Negre‐Salvayre A , Dousset N , Ferretti G , Bacchetti T , Curatola G , Salvayre R . Antioxidant and cytoprotective properties of high‐density lipoproteins in vascular cells. Free Radic Biol Med. 2006;41(7):1031‐1040. doi:10.1016/J.FREERADBIOMED.2006.07.006 16962927

